# Explorative study on scale cortisol accumulation in wild caught common dab (*Limanda limanda*)

**DOI:** 10.1186/s12917-022-03385-3

**Published:** 2022-08-22

**Authors:** Maaike Vercauteren, Bart Ampe, Lisa Devriese, Christel Palmyre Henri Moons, Annemie Decostere, Johan Aerts, Koen Chiers

**Affiliations:** 1 Department of Pathology, Bacteriology and Avian Diseases, Faculty of Veterinary Medicine, Ghent University, Salisburylaan 133, Merelbeke, Belgium; 2 Flanders Research Institute for Agriculture, Fisheries and Food (ILVO), Animal Husbandry, Scheldeweg 68, Melle, Belgium; 3 Flanders Marine Institute (VLIZ), InnovOcean site, Wandelaarkaai 7, Ostend, Belgium; 4 Department of Nutrition, Genetics and Ethology, Faculty of Veterinary Medicine, Ghent University, Heidestraat 19, Merelbeke, Belgium; 5 Stress Physiology Research GroupFlanders Research Institute for Agricultural and Fisheries and FoodDepartment of Biology, Faculty of Sciences, Animal Sciences Unit, Ghent University, Wetenschapspark 1, Ostend, Belgium

**Keywords:** Wild caught common dab (*Limanda limanda*), Chronic stress, Scale cortisol concentration, Hypercorticoid state

## Abstract

**Background:**

Flatfish live in a diverse marine ecosystem that is changing due to natural variations and anthropogenic influences. These changes can evoke a stress response mainly resulting in production of the glucocorticoid cortisol, which mediates effects on various levels of biological organization. The finding that cortisol accumulates in fish scales, offering a retrospective view on cortisol production, provides opportunities to use this matrix for chronic stress assessment. The present study is the first to gather information on scale cortisol concentration in wild-caught common dab (*Limanda limanda*), based on a two-pronged approach using (1) field measurements and (2) a laboratory in vivo-study where wild-caught dab were fed by cortisol-spiked feed during 30 or 90 days to demonstrate the possible accumulation of cortisol in the scales and to evaluate its impact on fish health.

**Results:**

Based on the field measurements, the average scale cortisol concentration in wild-caught fish was 0.0034 ± 0.0046 µg kg^−1^ scale (*n* = 67). This indicates that wild common dab is indeed able to incorporate cortisol in the scales.

Based on the experimental data, the cortisol-fed fish showed an increased plasma cortisol concentration (80.16 ± 82.58 µg L^−1^) compared to the control group (4.54 ± 9.57 µg L^−1^) after 30 days of cortisol feeding. The increase in plasma cortisol concentration was positively correlated with an increased cortisol concentration in the scale after 30 days of cortisol-spiked feeding. This correlation was, however, no longer observed after 90 days of cortisol-spiked feeding. Interestingly, cortisol concentration of the scales on the pigmented side was significantly higher compared to the non-pigmented side. Some health parameters such as epidermal thickness, body condition and *Ichthyobodo* sp. parasitic infection showed a correlation with scale cortisol concentration after 30 days.

**Conclusions:**

We have demonstrated that common dab is able to accumulate cortisol in its scales. This seems to occur proportionally to circulating concentrations of plasma cortisol in fish fed with cortisol supplemented feed after 30 days.

**Supplementary Information:**

The online version contains supplementary material available at 10.1186/s12917-022-03385-3.

## Background

Flatfish, as all marine organisms, live in a diverse ecosystem that is constantly changing due to natural variations and anthropogenic influences. Fish can perceive these changes as stressful stimuli. These stimuli can evoke a stress response, whereby an immediate production of catecholamines is followed by the production of glucocorticoid hormones produced by the hypothalamus-pituitary-interrenal (HPI) axis, resulting in the release of cortisol, the dominant glucocorticoid hormone in actinopterygian fishes, in the blood [3]. Cortisol is pleiotropic and mediates various physiological and metabolic pathways on cellular and tissue level (secondary response) that allow the fish to restore homeostasis and adapt to the new situation [14, 33]. However, if the perceived stressor is persistent, chronic stress can be evoked whereby physiologic response mechanisms are compromised. In this case, glucocorticoids lose their adaptive function and can have detrimental effects on the animals’ overall fitness and health and, as such, on the entire population due to growth reduction, decreased disease resistance and reproduction (tertiary response) [3].

The reaction of a fish to a stressful stimulus can be measured by analyzing the produced cortisol concentration, generally measured in blood plasma [3]. However, the sampling itself can cause an increase in circulating cortisol, thus hampering a clear conclusion [14, 23]. Because of this sampling limitation there is a need for accurate and validated analytical methods to quantify cortisol levels in a reliable and reproducible way, independent from sampling or handling of the fish. Cortisol was shown to accumulate in biological materials such as mammalian hair [6], avian feathers [18], whale baleen [15] and blubber [30]. In 2015, fish scales were found to accumulate cortisol over time and, as such, can provide a retrospective view on HPI axis (re)activity [1]. Cortisol incorporation in the scales was already demonstrated to be meaningful for chronic stress assessment in cultured goldfish (*Carassius auratus*) [19]; juvenile rainbow trout (*Oncorhynchus mykiss*) [8],juvenile milkfish (*Chanos chanos*) [13], common carp (*Cyprinus carpio*) [1], wild caught freshwater Catalan chub (*Squalius laietanus*) [9], European sea bass (*Dicentrarchus labrax)* [27] and two species of tropical tuna [25].

Flatfish species are generally accepted as a bio-indicator representing the health of the North Sea [, 7, 20]. At present, data on scale cortisol concentration are lacking in flatfish in the wild, while data on the latter could be useful in marine health monitoring programs*.* The quantification of chronic stress in a natural setting across a population can be indicative for a broader environmental challenge with possible effects on disease prevalence, reproduction and survival of the fish [23, 29]. Consequently, scale cortisol concentration (SCC) could be a reliable, easy-to-use ecological biomarker to be implemented in the general monitoring organized in the framework of the UN Sustainable Development Goals and the European Union Marine Strategy Framework Directive (MSFD).

The present study is the first to gather information on scale cortisol concentration in wild-caught common dab (*Limanda limanda*). To do so, a two-pronged approach was chosen based on (1) field measurements and (2) a laboratory in vivo-study. For collection of field data, measurements were performed on a small group of wild-caught common dab in order to establish initial indications on cortisol concentrations in the scales and the individual variation encountered in this species. Secondly, an in vivo study was conducted whereby wild-caught fish were fed with cortisol-spiked feed during 30 or 90 days to demonstrate the possible accumulation of cortisol in the scales and to evaluate its impact on fish health.

## Results

### Field measurements

All data from the field observations are provided in Table 1. The average scale cortisol concentration (SCC) observed in all fish (*n* = 67) was 0.0034 ± 0.0046 µg kg^−1^, with a minimum of 0.0006 µg kg^−1^ and maximum of 0.0322 µg kg^−1^. No significant difference was found between SCC in male (*n* = 21; 0.0029 ± 0.0023 µg kg^−1^) and female (*n* = 36; 0.0028 ± 0.0020 µg kg^−1^) fish (*p* = 0.8596).

**Table 1:** Overview of the fish characteristics from the field observations and their correlation with scale cortisol concentration. Mean ± sd are provided, minimal (min) and maximal (max) observations

	Length (cm)	Weight (g)	Body condition	Age	Weight per scale (mg)	Scale cortisol concentration (µg kg^−1^)
Mean	19.7 ± 3.1	76 ± 34	0.9 ± 0.2	3.4 ± 0.9	0.0778 ± 0.0621	0.00341 ± 0.0046
Min	12.5	14	0.4	2	0.02	0.000581
Max	28	174	1.6	5	0.42	0.032224
Correlation with scale cortisol concentration	Cor = 0.47 *p* < 0.0001	Cor = 0.43 *p* = 0.0004	Cor = -0.04 *p* = 0.7532	Cor = 0.33 *p* = 0.0605	Cor = 0.96 *p* < 0.0001	NA

*NA* Not applicable, *Cor* Pearson’s product-moment correlation coefficient

Length and weight were positively correlated with SCC (Length: cor = 0.47; *p* < 0.0001; weight: cor = 0.43; *p* = 0.0004), whereas for age a trend (cor = 0.33; *p* = 0.0605) was observed. Body condition was not correlated with SCC (cor = -0.04; *p* = 0.7532). The body mass of the fish was positively correlated to the weight of a scale (cor = 0.61, *p* < 0.0001). Healthy fish (*n* = 19), fish with skin lesions (*n* = 40) and fish with skeletal deformities (*n* = 8), showed no difference in SCC (*p* = 0.872).

### Laboratory in vivo study

#### Plasma cortisol analysis

**Fig. 1:**
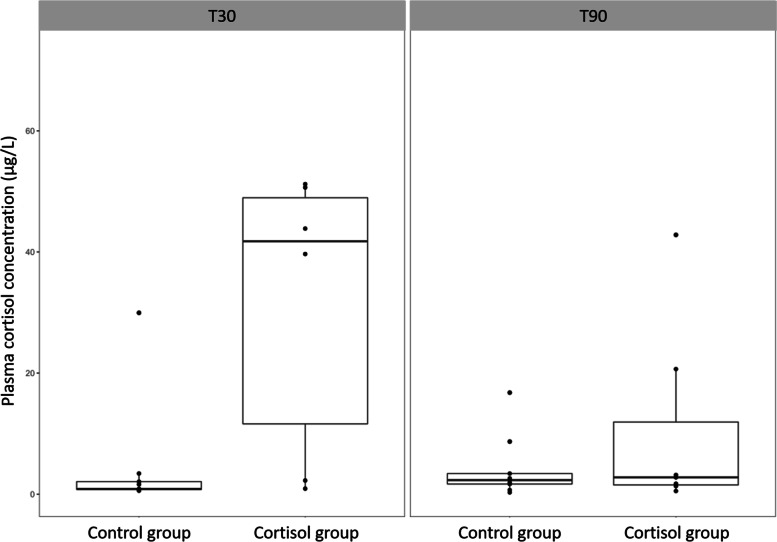
Plasma cortisol concentrations in control and cortisol groups after 30 (T30) and 90 (T90) days

At T30, fish of the control (CONT) group had significantly lower plasma cortisol concentration (PCC; 4.54 ± 9.57 µg L^−1^) compared to the cortisol-fed (CORT) group (80.16 ± 82.58 µg L^−1^) (*p* = 0.0164) (Fig. 1). Individual variation ranged from 0.57 µg L^−1^ to 255.23 µg L^−1^. At T90, PCC was 4.26 ± 5.29 µg L^−1^ and 37.99 ± 57.02 µg L^−1^ in CONT and CORT fish, respectively (*p* = 0.3513) (Fig. 1).

#### Scale cortisol analysis

**Fig. 2:**
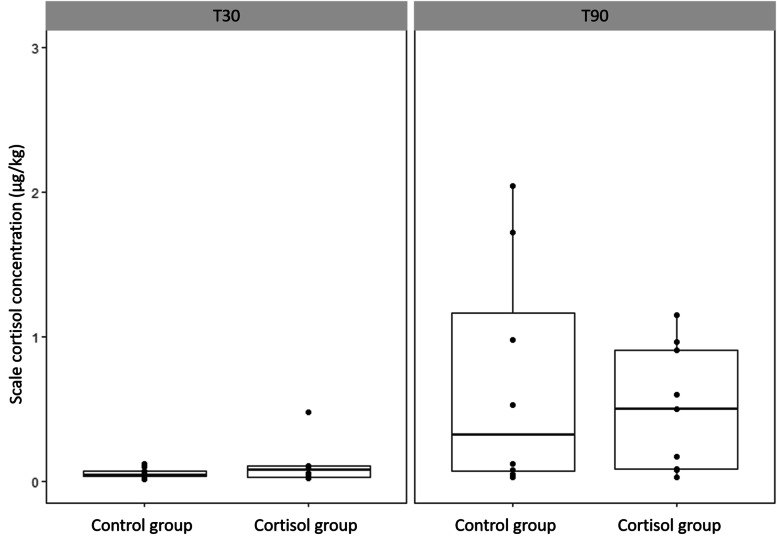
Scale cortisol concentrations in control and cortisol groups after 30 (T30) and 90 (T90) days. One extreme value of 4.93 µg/kg was removed from the CORT group at T90

**Fig. 3:**
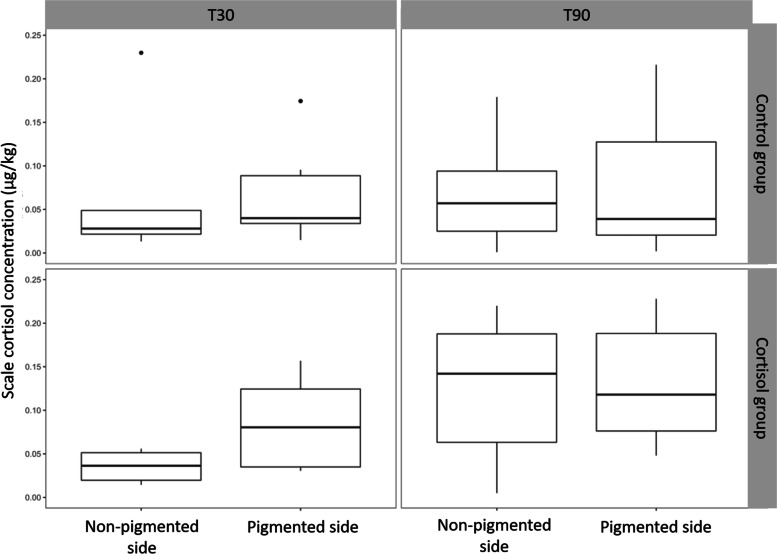
Scale cortisol concentration on pigmented and non-pigmented side

At T30, the average SCC was not significantly different in the CONT (0.06 ± 0.03 µg kg^−1^) and CORT (0.12 ± 0.15 µg kg^−1^) group (*p* = 0.4016) (Fig. 2). SCC increased over time in both CONT and CORT fish. This increase was only significant in CORT fish (*p* = 0.0356) and a trend was observed in CONT fish (*p* = 0.0907). At T90, no significant difference was observed between SCC of CONT (0.30 ± 0.38 µg kg^−1^) and CORT (0.50 ± 0.43 µg kg^−1^) group (*p* = 0.7317) (Fig. 2). The maximal SCC observed in the CORT group was 4.93 µg kg^−1^. Three fish of the CONT group that were sampled after 90 days had SCC values larger than 1.5 µg kg^−1^. The SCC on the pigmented side was significantly higher compared to the SCC on non-pigmented side (*p* = 0.0337). This observation was more pronounced in the CORT group at T30 (Fig. 3).

#### Correlation between scale and plasma cortisol concentration

At T30, a trend indicating a positive correlation between SCC and PCC was present in the CORT group (*p* = 0.0837), while fish of the CONT group showed no significant correlation between plasma and scale cortisol concentration (*p* = 0.4361). At T90, SCC was no longer correlated with PCC in the CORT fish (*p* = 0.7032) nor in the CONT fish (*p* = 0.1045).

### Effect on fish health: secondary and tertiary stress response correlated with scale cortisol concentration

A summary of the most important results is presented below. A more detailed description of the correlations between all measured parameters and the cortisol concentrations (both PCC and SCC) is provided in Additional file 2 including information on average values of all parameters as well as the statistical correlation with PCC and SCC at T30 and T90 for both groups (CORT and CONT).

#### Cellular and tissue effects: hepatic vacuolization, glycogen storage, goblet cells and thickness of epidermis

The degree of vacuolization and the amount of intra-hepatocellular glycogen present in the liver tissue did not show any correlation with the SCC at T30 and T90. Neither did number of goblet cells in the gills. The thickness of the epidermal skin tissue tended to be positively correlated with the SCC in CORT group at T30 (*p* = 0.0650), though not in the CONT fish (*p* = 0.8925). At T90, no significant correlations were observed.

#### Hematology: plasma osmolality, hematocrit and WBC count

No significant correlation was observed between SCC and the plasma osmolality, not in both groups and not at T30 nor at T90.No correlation between SCC and hematocrit value was observed, neither at T30 (CONT: *p* = 0.8475; CORT: *p* = 0.1433) nor at T90 (CONT: *p* = 0.4996; CORT: *p* = 0.1303). The SCC did not show any correlation with the ratio between WBC and RBC.

#### Effects on organism level: feeding behavior, growth, parasitic burden and histopathology

No correlations were observed between growth per day and PCC or SCC. A significant correlation was observed between the gained weight and SCC in the CORT fish at T30 (*p* = 0.0043). The evolution of the body condition was correlated with SCC at T30 in the CORT group (*p* = 0.0123). No correlations were observed between feeding response and SCC or PCC. Results of parasitological examination showed no conclusive results suggesting more infections in CORT groups compared to CONT groups, except for the presence of *Ichthyobodo sp*. in 7 out of 9 CORT fish, distributed over three experimental tanks and absent in the fish of the CONT groups.

Histological examination of the internal organs at T30 and T90 revealed no remarkable or consistent pathologies in CORT or in CONT fish (more information available in Additional file 2).

## Discussion

Fish scales have been proven to accumulate cortisol in time, hereby offering retrospective view on the HPI-axis (re)activity, in several species [1, 8, 9, 13]. The present study provides a first exploration on scale cortisol concentration and its correlation with plasma cortisol concentration in wild-caught common dab, a marine flatfish commonly used as a bio-indicator for marine health in the North Sea and adjacent areas [7, 20].

At first, based on field measurements, we explored whether common dab, as other teleost fish, was able to incorporate cortisol in their scales in time. This was indeed indicated by the field measurements where cortisol was detectable in the scales of most of the sampled common dab. The variability between fish was quite large but can be attributed to seasonal differences or changes in the sampling locations [9]. Furthermore, the link between various fish-specific characteristics such as length, weight, age, weight of the scales and scale cortisol concentration must be studied in detail to estimate their effect on the incorporation of cortisol in the scales. These field measurements, however, offer no insights in the mechanisms and the meaning of these scale cortisol concentrations. Therefore, ideally, a longitudinal study should be set up wherein repeated samples are collected per fish on a regular basis in time [10]. However, this would involve attributing repetitive lesions, thereby increasing risk for secondary infections and difficulties with maintaining the osmotic balance. We opted for a more ethically acceptable approach using a laboratory in vivo study.

In this laboratory study, cortisol supplementation via the feed was used to induce an increased plasma cortisol concentration in the fish [1, 2, 26]. The increased plasma cortisol concentration in fish fed with cortisol-spiked feed for 30 days suggests that this procedure was indeed effective. Importantly, as plasma cortisol concentration only provides a snapshot, it could be that during different times of the day, plasma cortisol concentrations were similar between both groups [21]. After 90 days in the experiment, the increased plasma cortisol concentrations were no longer observed. Various adaptive and/or regulatory processes such as a higher clearance rate from plasma, desensitization and habituation might have occurred. Also reduced feeding linked to reduced uptake of cortisol could explain this, but reduced feeding was not observed during analysis of the feeding response.

An increased plasma cortisol concentration might be regarded as a representative state for a stressed fish [3]. However, no information is available on the level and effects of chronic stress on common dab might be.

In our study, a correlation was found between plasma and scale cortisol concentration after 30 days of cortisol-spiked feeding, however, these results must be interpreted with care as only a small number of fish per group (*n* = 9) were analyzed. Nevertheless, this correlation indicates that an increased plasma cortisol concentration results in an increased scale cortisol concentration, a correlation that was hitherto only demonstrated in Catalan chub [9] and rainbow trout [8]. Moreover, this correlation was only observed in fish with increased plasma cortisol concentrations and not in control fish with lower plasma cortisol concentrations, which is in accordance with the findings in rainbow trout [8]. This is a first step in research on scale cortisol concentration in wild-caught common dab. The correlation between plasma and scale cortisol concentration was only observed after 30 days and not after 90 days, which is probably linked to decreased plasma cortisol concentrations after 90 days of cortisol-spiked feed, as mentioned before. More research, however, will be necessary to corroborate these results.

In this framework, scale cortisol concentration was observed to increase at population level between 30 and 90 days indicating that fish are accumulating cortisol in their scales over time. This is in accordance with some previously published studies [1, 13] but in contrast with others, whereby a decrease in scale cortisol concentration was observed [8, 19]. In the latter studies a different experimental designs were performed and different analytical methods to determine cortisol levels were used.

In Carbajal et al. [8], a continuous stressor, i.e. confinement with high densities, was used possibly resulting in the activation of various physiological processes such as habituation, desensitization and an increased metabolic clearance rate. Even an increased clearance from the scales might be possible [19]. It is conceivable that the daily recurring peak in circulating cortisol concentration, induced by feeding cortisol-spiked pellets, might have a different impact on the above mentioned biological processes in the fish in comparison with a continuous stressor. In addition, species-specific differences are also possible since responses to a certain stressor can depend on the type of stressor, duration, severity, (un)predictability, (un)controllability but also on the life history of an animal [29] and their genetic background [3].

When comparing to other teleost species, common dab has rather low concentrations of cortisol in both plasma and scales (Additional file 3), whereby the scale cortisol concentrations observed in common dab are comparable with those of milkfish [13]. However, they are considerably lower compared to those in chub, common carp, goldfish and rainbow trout, which range between 3 and 30 µg kg^−1^ in control fish [1, 8, 9, 19]. This difference is most likely species specific, and it emphasizes again the great gap of knowledge related to stress physiology in wild populations (Plankhurst 2011). Analytical methodological differences could also offer an explanation for the observed differences [1].

Another observation in our study was the difference between scale cortisol concentration in scales from pigmented and non-pigmented side, indicating that the pigmented side might accumulate more cortisol in its scales. The effect of pigment on cortisol accumulation is studied in mammalian hair whereby in some cases no effect is found [28] and in some cases clear differences in cortisol accumulation linked with hair color, whereby black hair had consistently lower cortisol concentration [4]. Effect of pigmentation on scale cortisol needs to be further explored. This needs to be taken into account during monitoring campaigns since the effect of diseases such as hypo-and hyperpigmentation are unknown and this urges for standardization during sampling.

In our study, a suite of health-related effects were evaluated on various levels of biological organization which were correlated to the observed cortisol concentration. Based on our data, a correlation was observed between plasma cortisol concentration and a decreased intrahepatocellular vacuolization in both cortisol-fed and control fish, indicating alterations in hepatic energy storage correlated with increased plasma cortisol concentration. In the control group, this could be the result from an increased glycogenolysis since the glycogen storage did also tended to be decreasing with increasing plasma cortisol concentration [14]. This was not observed in the cortisol-fed group, indicating rather a decrease in fat storage. Furthermore, the number of leukocytes in relation to the number of erythrocytes decreased and was negatively correlated to the plasma cortisol concentration in the cortisol-fed fish, potentially indicating a glucocorticoid mediated immunosuppressive effect. This was already reported [34] whereby even the intracellular killing ability of the neutrophils was reduced. These findings might be corroborated with an increased *Ichthyobodo* sp. infection in the gills of cortisol-fed fish after 30 days, which might indicate a lower disease resistance caused by the glucocorticoid mediated immunosuppressive effect. These correlations were not observed with scale cortisol concentrations. The thickness of the epidermal tissue and the gained weight seemed to be correlated with the scale cortisol concentration. The skin is not often reported as a glucocorticoid target tissue, but reports were made of ulceration development [14, 22, 31] and epithelial swelling [14, 16, 22, 31], amongst others, as an effect of stress. This was partly confirmed by the results of our study showing a correlation between scale cortisol concentration and thickness of the epidermal tissue under an increased scale cortisol concentration. This observation might be indicative for cell swelling or hyperplasia of the tissue. More research will be necessary for this result to be conclusive. The daily weight gain was also correlated with scale cortisol concentration indicating that fish that gained more weight showed a higher cortisol concentration in the scales, or the other way around. Importantly, the weight gain can be both the cause and the effect of the increased cortisol concentration. More research will be necessary to explain these results.

Based on our results, no clear and consistent indications were present correlating scale cortisol concentration to the analyzed health parameters in common dab. Various explanations are possible for this ranging from the existence of a latency phase for pathological effects or adaptation and/or desensitization to the increased cortisol concentration of fish. It could also indicate that regulatory factors are operative and allow the fish to deal with the increased circulation cortisol concentration. Furthermore, the sensitivity to stress is species specific [24]. Since this was the first experiment with wild caught common dab and taking into account that the pertinent literature lacks data on the stress response in this species in wild populations as well as in captivity, our findings emphasize the importance of a multidisciplinary approach including cellular, tissue, hematologic parameters and parameters on organism levels in stress related research.

## Conclusions

In conclusion, we have demonstrated that common dab is able to accumulate cortisol in its scales. This seems to occur proportionally to circulating concentrations of plasma cortisol in fish fed with cortisol supplemented feed after 30 days. In this study, no clinical health effects were correlated with increased cortisol concentration. However, due to the limited sample size, additional research is necessary to further explore and corroborate our results.

## Methods

To provide information on the scale cortisol concentration in common dab, a two-pronged approach was chosen based on (1) field measurements and (2) a laboratory in vivo-study. Methodology for both approaches is described separately below.

### Field measurements of scale cortisol concentration

In total, 67 common dabs were caught at various locations in the Belgian part of the North Sea in 2015 (*n* = 11), 2016 (*n* = 11), and 2017 (*n* = 45). More information on the campaigns and capture methods can be found in Vercauteren et al. [32]. Some fish showed an acute or healing skin lesion (*n* = 40) or skeletal deformity (*n* = 8). Following euthanasia (benzocaïne, 200 mg L^−1^), scales were scraped off from the pigmented side of the fish. The retrieved scales were rinsed with distilled water to remove mucus and other potential sources of exogenous glucocorticoids. Subsequently, scales were air-dried after which 50 scales were collected in an Eppendorf tube and frozen (- 20 °C) until analysis of the scale cortisol concentration (SCC) (see further). The sampling was carried out in accordance with the approved guidelines and legislation in force with regard to animal welfare.

### Laboratory in vivo study

#### Animals and housing

Thirty-six clinically healthy common dabs (20.9 ± 2.0 cm; 83.3 ± 27.5 g; age between 3 and 10 years) were caught in the Belgian part of the North Sea by the Research Vessel “Simon Stevin”. All fish (> 18 cm) were placed in a survival tank (1 × 1.2 × 1 m; 640 L) with continuous water renewal. Within four hours after capture, fish were transferred to the Marine Station of Ostend (MSO, Flanders Marine Institute – VLIZ) to large recirculating tanks (diameter 2.6 m, 4000 L) with sand covered bottoms (6 cm layer thickness, 0–2 mm grain size). During the acclimatization period at the MSO, water quality was monitored daily and water was renewed when needed to keep water parameters within preset ranges (pH 8.0 ± 0.5; 32 ± 1 practical salinity unit (PSU); 85 ± 5% oxygen saturation). Temperature was raised in due course (+ 1 °C in 48 h) from 9 ± 1 °C up to 16 ± 1 °C. Fish were fed with fresh whiting (*Merlangius merlangus*) and, after 10 days, feed was gradually switched to commercial feed (Efico Sigma 862, pellet size 4.5 mm, Biomar SAS, France). After an acclimatization period of three weeks, fish were transported (1 h) to the experimental tanks at the Faculty of Veterinary Medicine (Ghent University) using small transportation boxes (39.4 × 59.8 × 18.6 cm; 30 L) provided with an oxygen tablet (JBL GmbH & Co.KG, Germany).

Upon arrival at the experimental facility, fish were randomly distributed over six circular tanks (diameter 1.1 m; 800 L). The sex ratio in the tanks was similar. Tanks (six fish per tank) were filled with recirculating seawater and a sand layer (6 cm layer thickness, 0-2 mm grain size). Water quality was monitored daily, whereby throughout the experiment, following average values were recorded: 16.68 ± 0.48 °C; pH 8.97 ± 0.26; 31.36 ± 0.67 PSU; 82.17 ± 6.79% dissolved oxygen. Ammonia and nitrite levels never exceeded 0.2 and 1 mg L^−1^, respectively. Upon renewal of the water, artificial sea water was added (Instant Ocean, Aquarium Systems, USA). Due to presence of natural lighting, a natural day-night rhythm was provided as encountered between June and September.

**Fig. 4:**
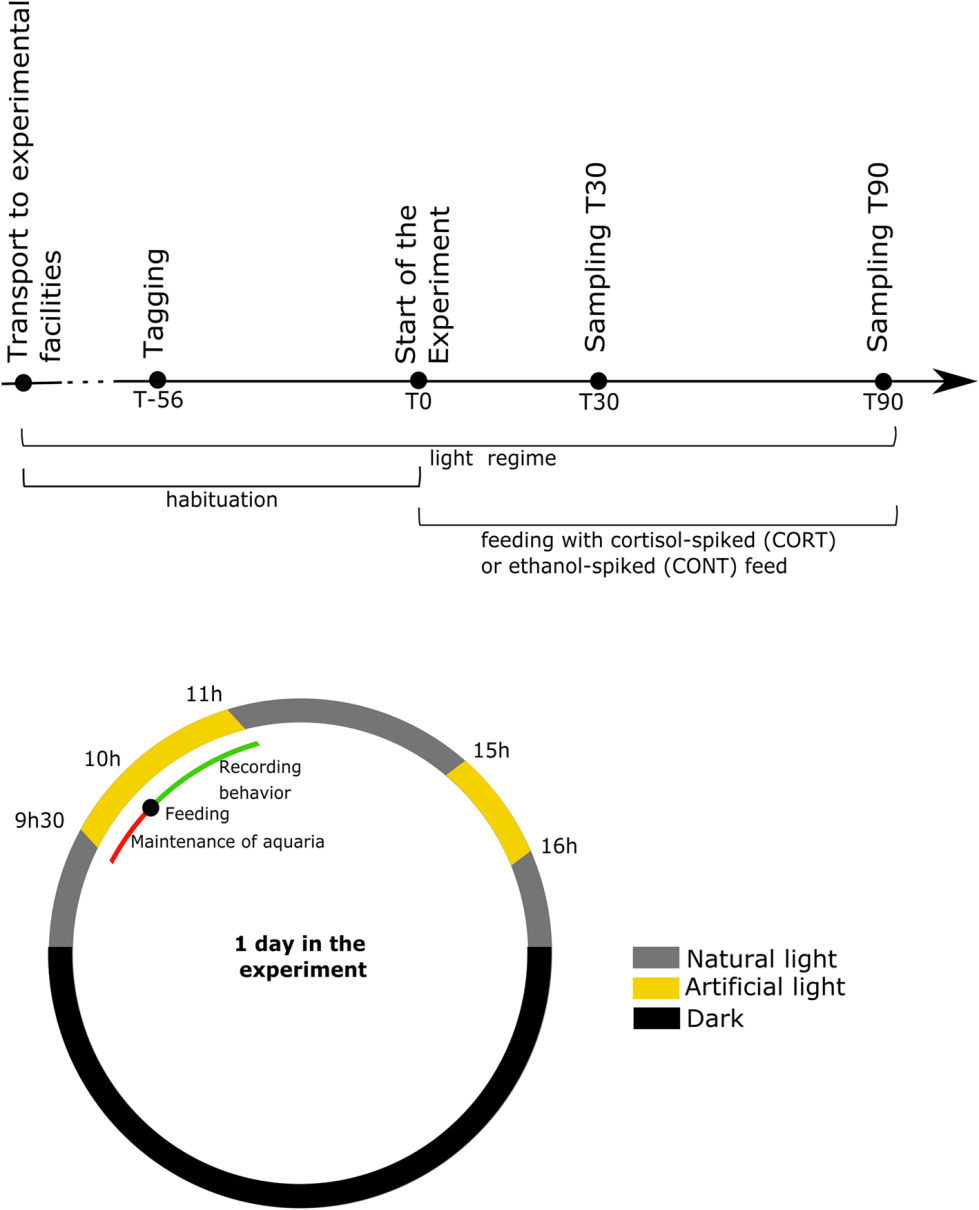
Overview of the experimental design. With a detailed schematic visualization of one day in the experiment. At each sampling point (T30 and T90), three fish per tank were sacrificed and multiple samples were collected

From the day of arrival at the experimental units until the end of the experiment, a strict daily routine was followed to allow optimal habituation of the fish (Fig. 4). All handlings (measurement of water quality parameters, renewal of the water, monitor mortality and morbidity) were limited to 30 min a day and were made predictable for the fish by a light cue (i.e. at 9:25 h, the light above every tank was lit, while between 9:30 h and 10 h, handlings were performed). Just prior to feeding, a camera was placed in the tanks to record feeding behavior. At 10 h, the fish were fed with commercially available pellets (Efico Sigma 862, pellet size 4.5 mm, Biomar SAS, France). The feeding response was recorded up to 1 h after feeding. Between 15 and 16 h the lights were lit again as mortality and morbidity were monitored.

The experimental design and housing was approved by the Ethical Committee of the Faculty of Veterinary Medicine and Bio-engineering Sciences, Ghent University (EC2017_92).

#### Experimental design

A schematic representation of the experimental design is provided in Fig. 4. Eight weeks (T-56) before the start of the experiment, all fish were anesthetized using Tricaine Methanosulfonate (MS-222; 100 mg L^−1^; Sigma Aldrich, Belgium). A T-bar anchor tag (Floy Tag Inc., USA) was inserted in the caudal epaxial musculature of all fish to allow individual identification. Hereby, all fish were photographed, weighed (W_B_), and measured (L_B_). After recovery, fish were replaced in their respective tanks where they could further habituate to the housing conditions and daily handling as described before.

At the start of the experiment (T0), three tanks were randomly assigned to the cortisol group (CORT) and three were assigned to the control group (CONT). The fish of the CORT group were fed with cortisol spiked feed (hydrocortisone, Sigma-Aldrich, USA) for the entire duration of the experiment (from T0 to T90). The spiked feed was prepared by uniformly spraying the pellets with cortisol dissolved in ethanol (1 mg mL^−1^) with a final concentration of 500 mg cortisol per kg feed, based on Aerts et al. [1]. Pellets were left to dry overnight at room temperature [1]. Fish of the CONT group were given the same amount ethanol-spiked feed without the addition of dissolved cortisol.

#### Sample collection

After 30 (T30) and 90 (T90) days, three fish per tank (i.e. nine fish per group) were euthanized using a stock solution of benzocaine (100 g benzocaine in 1 L ethanol) with a final concentration of 200 mg L^−1^ of seawater after which sampling was performed.

Blood was collected from each fish through caudal vessel lateral puncture using a heparinized needle and syringe. Time between catch and blood collection was on average 10 ± 4 min. Subsequently, all fish were measured (L_E_), weighed (W_E_) and pictures of pigmented and non-pigmented sides were collected. Fish were clinically inspected for the presence of skin lesions and/or deformities. Sagittal otoliths were obtained to determine the age of the fish (ICES, 2016). Wet mount preparations of gill biopsies and skin mucus samples were collected for parasitological examination. Scales from both the pigmented and non-pigmented side were collected and immediately frozen (- 20 °C) until cortisol analysis (see further).

Finally, a necropsy was performed for gross examination of internal organs. For histological examinations, samples of the gill, skin, liver, spleen, kidney, heart and intestines were collected. Formalin-fixed tissues were processed according to standard techniques, sectioned (5 µm) and stained with haematoxylin and eosin (H & E) or Periodic Acid Shiff (PAS).

#### Plasma and scale cortisol analysis

Heparinized tubes filled with blood were centrifuged (10 000 × *g* for 5 min at 5 °C). Plasma was collected and plasma cortisol concentration (PCC) was quantified using ultra-performance liquid chromatography coupled to tandem mass spectrometry (UPLC-MS/MS), derived from [1].

Scale cortisol concentration was quantified as described by Aerts and colleagues [1]. Briefly, after defrosting, mucus was removed, the scales were cut in smaller pieces and further homogenized using PowerBead tubes (ceramic 2.8 mm, Qiagen) and a bead ruptor (PowerLyzer 24, Qiagen) after which cortisol was extracted with methanol (VWR international BVBA, Belgium). After vortex-mixing and centrifugation (10 min, 3500 × *g*), the supernatant was collected and evaporated under nitrogen at 60 °C using a Turbovap nitrogen evaporator (Biotage, Sweden). The dried pellet was re-suspended and purified using a Gracepure™ C_18_ solid-phase extraction column after which UPLC-MS/MS (Xevo TQS, Waters, Millford, USA) analysis was performed.

### Effect on fish health: secondary and tertiary responses correlated with increased cortisol concentration

#### Cellular and tissue response

Based on the histological samples of liver, gills and skin, cell and tissue related parameters were studied. As a proxy for metabolic effects, the degree of intrahepatocellular vacuolization in the liver was quantified based on H & E staining. An additional PAS staining was performed staining glycogen present in the vacuoles [35]. Five images of the stained histological sections were randomly acquired. The uncolored (H & E staining – lipid and/or glycogen storage) or purple (PAS staining – glycogen storage) area was calculated on each image using scientific image analysis software Image J (version 1.4). Based on a PAS staining of the gills, the number of goblet cells (GC) in gill filaments and lamellae were counted in five random gill filaments per fish. The results were expressed as the number of GC per 100 µm. The thickness of the epidermis was measured at 25 random chosen places based on H & E stained tissue samples using Image J software. To avoid bias during the assessment, image origins were blinded from the observers.

#### Hematology

Blood samples were used to estimate the osmotic balance and changes in hematological parameters. Plasma osmolality was determined using an osmometer (Osmomat 030, Gonotec, Berlin, Germany). For measuring hematocrit, the blood was dispensed in heparin containing micro-hematocrit tubes (GMBH + CO KG, Wertheim, Germany) and were centrifuged (10,000 g; 5 °C; 5 min). A blood smear was prepared using approximately seven µL of blood quickly after sampling. The smear was dried and stained using Hemacolor rapid staining (VWR, Belgium). The ratio between the total white blood cell (WBC) count and the total amount of red blood cells (RBC) was determined as a proxy for the immunological state. To estimate the WBC and RBC counts, blood smears were analyzed under the microscope and five pictures were collected per smear. Per picture, the number of WBC per 100 RBC was counted in three random fields. To avoid bias, the image origins were blinded from the observer.

#### Effects on organism level

Effects on organism level were evaluated using growth rate, body condition, feeding response (i.e., the total number of pellets eaten) and general health of the fish. The growth per day was calculated as the L_E_ minus L_B_ divided by the number of days the fish was in the experiment (30 or 90 days). The daily weight gain was calculated in a similar way (W_E_-W_B_). The Fulton condition index (K = 100*(W/L^3^) [12] was calculated at T-56 (K_B_) and at sampling (T30 or T90) (K_E_). Individual feeding response, measured as the number of pellets eaten per fish, was quantified using the video footage collected during feeding (10 h – 11 h). The general health of the fish was assessed by determining the presence of parasites based on the gill biopsies and skin mucus samples. In addition, gross and histological lesions in the internal organs were examined.

### Statistical analysis

#### Statistical analysis of field measurements

Normality was evaluated using a QQ plot and, when needed, a logarithmic transformation of the data was performed to obtain normality. The difference between SCC in males and females was tested using a Welch two sample t-test. The correlation between the length, condition, weight, age, weight per scale, and SCC was studied using a Pearson’s product-moment correlation. The difference in SCC in fish with no lesions, skeletal deformities or skin ulcerations was tested using an ANOVA. Differences were considered to be significant when *p*-values were lower than 0.05. A *p*-value between 0.05 and 0.1 was considered a trend.

#### Statistical analysis of laboratory in vivo study

Normality was evaluated based on graphical evaluation (QQ plot) and, when needed, a logarithmic transformation of the data was performed to obtain normality. To ensure valid comparison between CONT and CORT fish, differences in fish-related characteristics (length, weight, condition, age) were analyzed using a linear mixed model (proc GLIMMIX) with tank as a random intercept. No differences were observed (Additional file 1) therefore allowing comparison between both groups without taking into account differences in fish-related characteristics.

The correlation between SCC and PCC was analyzed using a linear mixed model (proc GLIMMIX). All analyses were stratified by sampling day (T30/T90) and by group (CONT/CORT). The average SCC per fish was used calculated as the average of SCC of pigmented and non-pigmented side, unless specified otherwise.

Correlations between PCC, SCC and specified cellular, tissue, osmotic, hematological and whole-organism parameters were studied using a linear mixed model (proc GLIMMIX). SCC and PCC were used as response variables while other parameters were used as variables. All analyses were stratified by sampling day (T30/T90) and by group (CONT/CORT). The presence of parasites in skin or gills or gross and/or histological lesions in gills and internal organs was analyzed in a descriptive manner.

In all models, tank replicate was included as a random intercept. Differences were considered to be significant when *p*-values were lower than 0.05. A *p*-value between 0.05 and 0.1 was considered a trend. However, due to the low amount of fish sampled per group per sampling point (*n* = 9), *p* values below 0.1 might be suggestive of a significant difference although drawing clear conclusion would need more evidence. All statistical analyses were performed using SAS 9.4, graphical representations were constructed in R Studio.

## Supplementary Information

**Additional file 1:** Mean fish characteristics before the experiment of the control group (CONT) and fish that were fed with feed supplemented with cortisol (CORT). Length (*p* = 0.6211), weight (*p* = 0.8491) and body condition (*p* = 0.1810) of the common dab were not significantly different between CORT and CONT fish. Fish of both groups did not differ in age (*p* = 0.4582). The sex ratio of both groups were similar with 6 (33 %) females and 12 (67 %) males in the CONT group and 7 (39 %) females and 11 (61 %) males in the CORT group (*p* = 0.4582).

**Additional file 2:** Detailed overview of the effects on fish health correlated with plasma (PCC) and scale cortisol concentrations (SCC).

**Additional file 3:** Overview of the existing research and reported values of scale and plasma cortisol concentrations and the methods used.

## Data Availability

The datasets generated during and/or analyzed during the current study are available from the corresponding author on reasonable request.

## Abbreviations

CONT: Control group
CORT: Cortisol-fed group
H&E: Haematoxylin and eosin
HPI: Hypothalamic-pituitary-interrenal
HSC: Hypothalamic-sympathetic-chromaffin cell
K_B_: Body condition at the beginning of the experiment
K_E_: Body condition at the end of the experiment
L_B_: Length at the beginning of the experiment
L_E_: Length at the end of the experiment
MS-222: Tricaine Methanosulfonate
MSFD: Marine Strategy Framework Directive
PAS: Periodic Acid Shiff
PCC: Plasma cortisol concentration
RBC: Red blood cell
SCC: Scale cortisol concentration
UPLC- MS/MS: Ultra-performance liquid chromatography coupled to tandem mass spectrometry
W_B_: Weight at the beginning of the experiment
WBC: White blood cell
W_E_: Weight at the end of the experiment
