# Supplementary material for: Explorative study on scale cortisol accumulation in wild caught common dab (Limanda limanda)
Source: BMC Vet Res. 2022 Aug 22;18:324. doi: 10.1186/s12917-022-03385-3 (PMC9394017; doi:10.1186/s12917-022-03385-3)
Supplement: Supplementary file 1 — Additional file 1: Mean fish characteristics before the experiment of the control group (CONT) and fish that were fed with feed supplemented with cortisol (CORT). Length (p = 0.6211), weight (p = 0.8491) and body condition (p = 0.1810) of the common dab were not significantly different between CORT and CONT fish. Fish of both groups did not differ in age (p = 0.4582). The sex ratio of both groups were similar with 6 (33 %) females and 12 (67 %) males in the CONT group and 7 (39 %) females and 11 (61 %) males in the CORT group (p = 0.4582). [file 12917_2022_3385_MOESM1_ESM.pdf]

Additional file 1 Mean fish characteristics before the experiment of the control group (CONT) and fish that were fed with feed supplemented with cortisol (CORT). Length (p = 0.6211), weight (p = 0.8491) and body condition (p = 0.1810) of the common dab were not significantly different between CORT and CONT fish. Fish of both groups did not differ in age (p = 0.4582). The sex ratio of both groups were similar with 6 (33 %) females and 12 (67 %) males in the CONT group and 7 (39 %) females and 11 (61 %) males in the CORT group (p = 0.4582).

Group	N	Length (cm)	Weigth (g)	Body condition	Age (years)	Sex ratio
**CONT**	18	20.7 ± 1.7	82.4 ± 25.2	0.9 ± 0.1	5 ± 2	6 F / 12 M
**CORT**	18	21.1 ± 2.2	84.3 ± 30.4	0.9 ± 0.1	5 ± 2	7 F / 11 M
**All fish**	36	20.9 ± 2.0	83.3 ± 27.5			13 F / 23 M

N: number of fish in each group; F= female; M= male
