# Supplementary material for: Explorative study on scale cortisol accumulation in wild caught common dab (Limanda limanda)
Source: BMC Vet Res. 2022 Aug 22;18:324. doi: 10.1186/s12917-022-03385-3 (PMC9394017; doi:10.1186/s12917-022-03385-3)
Supplement: Supplementary file 2 — Additional file 2: Detailed overview of the effects on fish health correlated with plasma (PCC) and scale cortisol concentrations (SCC). [file 12917_2022_3385_MOESM2_ESM.pdf]

Additional file 2 Detailed overview of the effects on fish health correlated with plasma (PCC) and scale cortisol concentrations (SCC).

Effects on fish health: secondary and tertiary stress response correlated with scale cortisol concentration

A detailed description of the correlations between all measured parameters and the cortisol concentrations (both PCC and SCC) is provided here including information on average values of all parameters as well as the statistical correlation with PCC and SCC at T30 and T90 for both groups (CORT and CONT).

Average values of all parameters as well as the statistical correlation with PCC and SCC at T30 and T90 for both CORT-CONT groups are provided in Additional file 3.

Cellular and tissue effects

Detailed results of effects on cellular and tissue level and the correlation with PCC and SCC are provided in Table S21.

At T30, the degree of vacuolization was negatively correlated with the PCC of the CONT fish ($p = 0.0605$), and a significant positive correlation was observed in the CORT group ($p = 0.0092$). The amount of intra-hepatocellular glycogen present in the liver, showed a significant negative correlation with PCC in the CONT group after 30 days ($p = 0.0269$), while SCC was not significantly correlated with the degree of vacuolization nor with the glycogen content (CONT: $p = 0.2373$; CORT: $p = 0.4366$). At T90, no significant correlations were observed, neither with the PCC nor with SCC.

In the gills, no significant correlation was observed between PCC or SCC and the total number of goblet cells neither at T30 nor at T90.

No significant correlation was observed between the thickness of the epidermal tissue and the PCC, neither at T30 nor at T90 and in neither group. Thickness of the epidermal skin tissue tended to

be positively associated with the SCC in CORT group at T30 (p = 0.0650), though not in the CONT fish (p = 0.8925). At T90, no significant correlations were observed.

Table S1: Overview of p-values of correlations between plasma scale cortisol (PCC) or scale cortisol concentrations (SCC) and effects on cellular and tissue level. Mean and standard deviation are indicated for each group and each sampling point. Significant (p < 0.05) or trending (p < 0.1) correlations are highlighted in blue.

Hepatic vacuolization

Group	Sampling day	Mean	SD	Correlation with PCC	Correlation with SCC
Control	T30	0.42	0.11	0.0605	0.2373
Cortisol	T30	0.44	0.12	0.0092	0.4366
Control	T90	0.35	0.09	0.5789	0.8114
Cortisol	T90	0.38	0.08	0.1812	0.9345

Glycogen storage

Group	Sampling day	Mean	SD	Correlation with PCC	Correlation with SCC
Control	T30	0.33	0.17	0.0269	0.5796
Cortisol	T30	0.31	0.11	0.3642	0.9228
Control	T90	0.34	0.15	0.5243	0.5616
Cortisol	T90	0.22	0.16	0.4095	0.1587

Number of goblet cells in gills

Group	Sampling day	Mean	SD	Correlation with PCC	Correlation with SCC
Control	T30	41.25	6.72	0.7479	0.7118
Cortisol	T30	32.80	13.22	0.6538	0.1898
Control	T90	43.88	9,04	0.5432	0.6677
Cortisol	T90	54.82	14.71	0.8102	0.2774

Thickness of epidermis

Group	Sampling day	Mean	SD	Correlation with PCC	Correlation with SCC
Control	T30	90.77	31.48	0.2429	0.8925
Cortisol	T30	54.71	17.02	0.4594	0.065
Control	T90	75.06	22.13	0.2225	0.158
Cortisol	T90	52.17	7.92	0.343	0.131

Hematology

Detailed results of effects on hematology and the correlation with PCC and SCC are provided in Table S2.

No significant correlation was observed between PCC or SCC and the plasma osmolality, not in both groups and not at T30 nor at T90.

Hematocrit tended to be correlated with the PCC in CONT fish at T30 (p = 0.0560), but not in CORT fish (p = 0.1832). At T90 no correlations were observed (CONT: p = 0.6333; CORT: p = 0.5690). No correlation between SCC and hematocrit value was observed, neither at T30 (CONT: p = 0.8475; CORT: p = 0.1433) nor at T90 (CONT: p = 0.4996; CORT: p = 0.1303).

Changes in inflammatory status, determined by the ratio of WBC to RBC, showed a negative correlation with PCC in the CORT group on T30 (p = 0.0180). The correlation was no longer observed on T90 (p = 0.5690) and neither in the CONT group on T30 (p = 0.3533) or T90 (p = 0.3503). The SCC did not show any correlation with the ratio between WBC and RBC.

Table S2: Overview of p-values of correlations between plasma scale cortisol (PCC) or scale cortisol concentrations (SCC) and hematology. Mean and standard deviation are indicated for each group and each sampling point. Significant (p < 0.05) or trending (p < 0.1) correlations are highlighted in blue.

Osmolality

Group	Sampling day	Mean	SD	Correlation with PCC	Correlation with SCC
Control	T30	352.00	15.00	0.4026	0.202
Cortisol	T30	358.00	7.70	0.9442	0.4336
Control	T90	347.67	15.59	0.7923	0.1831
Cortisol	T90	344.67	11.67	0.2001	0.7742

Hematocrit

Group	Sampling day	Mean	SD	Correlation with PCC	Correlation with SCC
Control	T30	24.78	6.04	0.056	0.8475
Cortisol	T30	24.44	7.39	0.1832	0.1433
Control	T90	22.00	5.41	0.6333	0.4996
Cortisol	T90	28.11	7.32	0.569	0.1303

White vs. Red blood cell ratio

Group	Sampling day	Mean	SD	Correlation with PCC	Correlation with SCC
Control	T30	0.02	0.00	0.3533	0.1836
Cortisol	T30	0.03	0.01	0.018	0.4457
Control	T90	0.04	0.02	0.3503	0.3861
Cortisol	T90	0.06	0.06	0.2803	0.3045

Effects on organism level

Detailed results of effects on organism level and the correlation with PCC and SCC are provided in Table S3.

No correlations were observed between growth per day and PCC or SCC. No correlation was observed between PCC and the gained weight, neither at T30 nor at T90. A significant correlation was observed between the gained weight and SCC in the CORT fish at T30 ($p=0.0043$). The evolution of the body condition was correlated with SCC at T30 in the CORT group ($p = 0.0123$). No correlations were observed between feeding response and SCC or PCC.

Parasitological examination revealed that seven fish (CONT: n = 4, CORT: n = 3) had *Epitheliocystis* sp. in the gills. No parasites were detected on skin of the fish.

Histological examination of the gills of fish at T30 revealed the presence of *Ichthyobodo* sp. in seven out of nine CORT fish, distributed over the three tanks. This ectoparasitic flagellate was present in different numbers and affected gill filaments causing slight hypertrophy and 'ruffled' appearance. *Ichthyobodo* sp. was not observed in gills of CORT fish at T90, neither in CONT fish at T30 and T90. Goblet cell hyperplasia was observed in CONT (n = 3) and CORT (n = 1) fish.

Histological examination of the internal organs at T30 and T90 revealed no remarkable or consistent pathologies in CORT or in CONT fish. The liver of two CORT fish showed signs of a pre-tumor phase as hyperplasia of hepatocytes were detected. In the kidneys, two fish (one CORT, one CONT) showed nephrocalcinosis, one CORT fish showed thyroidization of the kidney tubuli. The spleen did not show any abnormalities. Signs of myocarditis were found in one CONT fish. In the intestinal wall of nine fish (CONT (n = 4) and CORT (n = 5)) encapsulated parasites were detected.

Table S3: Overview of p-values of correlations between plasma scale cortisol (PCC) or scale cortisol concentrations (SCC) and the fish health on organism level. Mean and standard deviation are indicated for each group and each sampling point. Significant (p < 0.05) or trending (p < 0.1) correlations are highlighted in blue.

Growth per day

Group	Sampling day	Mean	SD	Correlation with PCC	Correlation with SCC
Control	T30	0.031	0.024216105	0.4173	0.1489
Cortisol	T30	0.026	0.025153848	0.3409	0.1647
Control	T90	0.015	0.010310675	0.5929	0.8466
Cortisol	T90	0.010	0.00822981	0.7058	0.3646

Weigth gain per day

Group	Sampling day	Mean	SD	Correlation with PCC	Correlation with SCC
Control	T30	1.167	0.564210363	0.2203	0.2262
Cortisol	T30	1.185	1.02998262	0.1897	0.0043
Control	T90	0.398	0.271471628	0.3257	0.8118
Cortisol	T90	0.419	0.143694635	0.9651	0.1762

Evolution of condition

Group	Sampling day	Mean	SD	Correlation with PCC	Correlation with SCC
Control	T30	0.002	0.000898765	0.2067	0.7651
Cortisol	T30	0.002	0.00163936	0.1876	0.0123
Control	T90	0.002	0.001317194	0.3323	0.6098
Cortisol	T90	0.002	0.001190355	0.6957	0.4925

Feeding response

Group	Sampling day	Mean	SD	Correlation with PCC	Correlation with SCC
Control	T30	1.000	1.587	0.4359	0.6374
Cortisol	T30	0.712	1.205	0.4786	0.467
Control	T90	1.319	1.920	0.953	0.1329
Cortisol	T90	2.792	3.140	0.8166	0.2966
