# Supplementary material for: Explorative study on scale cortisol accumulation in wild caught common dab (Limanda limanda)
Source: BMC Vet Res. 2022 Aug 22;18:324. doi: 10.1186/s12917-022-03385-3 (PMC9394017; doi:10.1186/s12917-022-03385-3)
Supplement: Supplementary file 3 — Additional file 3: Overview of the existing research and reported values of scale and plasma cortisol concentrations and the methods used. [file 12917_2022_3385_MOESM3_ESM.pdf]

Additional file 3 Overview of the existing research and reported values of scale and plasma cortisol concentrations and the methods used.

Species	Control/stressed	Cortisol level in scales (ng/g)	Used method	Cortisol level in plasma (ng/ml)	Method	Reference
Milkfish		0.002	UPLC-MS/MS	/	/	Hanke et al.
Goldfish	Control	± 6	RIA	low	RIA	Laberge et al., 2019
	Acute stress	± 5	RIA	200	RIA	
	Chronic stress	± 20-30	**RIA**	200	RIA	
Catalan chub (wild)	Presumably seasonal variation	± 25-40	EIA	± 300-500	EIA	Carbajal et al., 2019
Rainbow trout	Chronic stress	± 3	EIA	± 400	EIA	Carbajal et al., 2019
Common carp	control	3	UPLC-MS/MS	59.41	RIA	Aerts et al., 2015
	stressed	10	UPLC-MS/MS	35.4	RIA	
	cortisol-fed	6	UPLC-MS/MS	863.95	RIA	
common dab (wild)	Control	0.003 ± 0.005	UPLC-MS/MS	/	/	This study
common dab (wild in in vivo experiment)	Control	0.057 ± 0.059 (T30)	UPLC-MS/MS	4.54 ± 9.57	RIA	
	Cortisol-fed	0.118 ± 0.168 (T30)	UPLC-MS/MS	80.16 ± 82.58	RIA	
